# Green space exposure predicts manic episodes in bipolar disorder: one year longitudinal analysis using smartphone-derived GPS data

**DOI:** 10.1038/s44184-026-00242-1

**Published:** 2026-09-24

**Authors:** Marvin Guth, Carl A. Bittendorf, Clemens Krug, Esther Mühlbauer, Wolfram E. Severus, Michael Bauer, Markus Reichert, Vera M. Ludwig, Ulrich W. Ebner-Priemer

**Affiliations:** 1 Faculty of Sport Science, Department of eHealth and Sports Analytics, Ruhr-University Bochum, Bochum, Germany; 2 Mental mHealth Lab, Institute of Sports and Sports Science, Karlsruhe Institute of Technology, Karlsruhe, Germany; 3 Department of Psychiatry and Psychotherapy, University Hospital Carl Gustav Carus, Technical University Dresden, Dresden, Germany; 4 Faculty of Medicine Carl Gustav Carus, Technical University Dresden, Dresden, Germany; 5 Asklepios Klinik Nord-Ochsenzoll, Hamburg, Germany; 6 Department of Sport and Exercise Science, Faculty of Natural and Life Sciences, University of Salzburg, Salzburg, Austria; 7 Department of Psychiatry and Psychotherapy, Central Institute of Mental Health, University of Heidelberg, Medical Faculty Mannheim, Mannheim, Germany

**Keywords:** Diseases, Health care, Psychology, Psychology, Risk factors

## Abstract

Urban living is linked to increased psychiatric morbidity, while green spaces may protect mental health via stress-buffering effects. Yet, the role of green space exposure in the onset of affective episodes in bipolar disorder (BD) remains unclear. Using 12 months of GPS data from 29 participants with BD-I/II, we quantified daily exposure to green spaces (green area & NDVI; covariates: population density & imperviousness). Affective status (euthymia, depressive or (hypo)manic episode) was assessed via SCID-based biweekly clinician interviews (26 assessments per participant). Multilevel logistic regression examined moving averages and temporal variance of green space exposure during prodromal and episode weeks relative to euthymia. Reduced mean green space exposure and increased exposure variability were consistently associated with prodromal phases of (hypo)manic episodes, with variance effects persisting into acute (hypo)mania. In contrast, depressive episodes showed limited prodromal predictability. These findings suggest dynamic environmental signatures consistent with early-warning dynamics as modifiable contextual correlates in BD.

## Introduction

Meta-analytic evidence reveals that urban living is associated with increased rates of psychiatric disorders^1–3^, especially for mood disorders^3^. Concurrently, green spaces have increasingly been recognized as protective factors mitigating psychiatric morbidity^4,5^, and gained considerable attention in popular science discourse (‘forest bathing'^6–8^). A recent meta-analysis^9^ addressed this by identifying urban green spaces as crucial factors in reducing negative emotional states such as depression and anxiety, while simultaneously promoting overall mental well-being.

Several theoretical frameworks have been proposed to explain how exposure to green spaces may exert a calming and restorative effect on individuals. The *biophilia hypothesis* suggests that humans possess an innate affinity for nature, underscoring our species’ deep-rooted connection to natural environments^10^. The *stress reduction theory* posits that natural settings reduce physiological stress responses, leading to lower heart rates and decreased muscle tension^11^. Additionally, the *attention restoration theory* proposes that the sensory qualities of nature, such as its visual and auditory stimuli, help restore attentional capacity and mitigate cognitive fatigue^12^.

As the reported theories assume momentary associations, green space exposure may also influence the fluctuation of psychopathological symptoms significantly. Adopting such a perspective would broaden the scope of the green space exposure effect beyond the mere cross-sectional, odds ratio-based distinction between cases and controls. A particularly interesting disorder in this regard is bipolar disorder (BD). BD affects up to 40 million people worldwide, poses substantial personal and societal burdens^13,14^, and BD management aims to reduce the frequency, intensity, and duration of affective episodes to prolong periods of remission^15^. Given that stress is shown to be a well-established trigger for episode onset and relapse in BD, as well as a factor contributing to episode severity and impaired functioning^16^, investigating the interaction of environmental exposure with episode occurrence appears particularly promising.

Fortunately, intensive longitudinal methods such as digital phenotyping and ecological momentary assessment (EMA) offer an opportunity to monitor symptoms, behavior, and environmental context in real time^17,18^. By integrating GPS-based mobility data, these approaches can quantify individual exposure to urban versus natural environments, enabling a direct investigation of how daily interactions with green spaces influence the onset and course of affective episodes in BD^19^.

Three studies have examined location-based digital phenotyping for BD prediction^20–22^. Common among all of them is that they derived mobility pattern from GPS data, such as number of places visited or transition times. However, they did not investigate characteristics of the environment exposed, such as green spaces, population density, imperviousness, etc. In detail, Palmius et al.^20^ assessed three months of geographic data from 22 BD participants and 14 healthy controls (HC), showing the usefulness of entropy- and home stay-related mobility features in predicting depressive symptomatology in BD. Faurholt-Jepsen et al.^21^ collected smartphone location data from 48 BD patients and 31 healthy controls over nine months, showing associations between location based data and self-ratings of depression. Using the BipoSense dataset, Ludwig et al.^22^ applied activity related parameters, which were partially GPS-derived, to predict prodromal symptoms of emerging affective episodes in 28 patients with BD, which were tracked over 12 months. In sum, none of these studies investigated the characteristics of the exposed environment. Additional limitations are short study duration and therefore a limited variance of symptomatology within participants^20^, self-reported symptom changes instead of expert rated disease states^20,21^as ground truth and relating mobility pattern to current symptomatology instead of investigating prodromal episodes. This is crucial, as accurate prediction of emerging affective episodes is key to enabling early interventions with the aim to reduce the intensity or duration of emerging episodes or ideally prevent them altogether.

Alongside these intensive longitudinal and digital phenotyping approaches, dynamical systems theory^23–25^ (DST) provides a complementary framework for understanding the emergence of affective episodes. DST proposes that critical transitions in complex systems are preceded by specific changes in system-specific measures, so-called early warning signs. DST highlights stability, transitions, and critical thresholds, allowing early detection of shifts between manic and depressive states through changes in intra-individual variability, which can be statistically observed as increased variance in system-specific measures^26–29^.

Building on epidemiological evidence linking urbanicity and limited green space exposure to heightened indices of psychiatric disorders, and models which postulate green spaces exposure as a stress buffer, thereby affecting the emergence of new illness episodes, we examined daily urban exposure in a sample of individuals with BD. We thus expected that lower green space exposure is associated with a higher likelihood of emerging affective episodes in BD due to the stress buffering effect (Hypothesis 1; H1**)**. Accordingly, we hypothesized that reduced green space exposure indices would increase the probability of prodromal weeks preceding both depressive and manic episodes. Additionally, we conducted exploratory analyses to investigate whether observed associations persist beyond the prodromal phase and remain detectable during later stages of affective episodes. Moreover, we examined whether the observed effects were primarily driven by the beneficial influence of green space exposure or, alternatively, by the relative absence of urbanicity by considering population density and exposure to impervious surfaces.

Our second hypothesis (H2) leverages DST to examine whether increased within-person variability (14-day moving variance) of green space exposure, as a dynamical index of DST, is predictive for prodromal phases. Analogous to our first hypothesis, we conducted additional exploratory analyses examining affective episodes and other environmental indicators, such as population density and exposure to impervious surfaces.

Tests of H1 and H2 focused on prodromal weeks (early and late) and on green space metrics (green area and NDVI) as theory driven confirmatory analyses. All analyses extending (i) beyond prodromes into episode weeks, and/or (ii) to other environmental indicators (population density, imperviousness) were treated as exploratory.

## Methods

### Sample

We analyzed data from the BipoSense study^22,30,31^, which combines 12 months of continuous passive and active sensing with bi-weekly expert interviews (26 interviews per participant). Patients (BD I/II) were recruited from a specialized outpatient clinic from November 2014 to June 2016 and met strict diagnostic criteria, including ≥3 affective episodes in the past five years, ensuring the inclusion of patients with a higher risk for emerging episodes during the study period. Affective status was assessed using SCID-I (DSM-4)^32^ for the prior 14 days. The prodromal phase was operationalized as the 14 days preceding the clinically defined onset of a depressive or (hypo)manic episode, based on SCID-I assessments. This 14-day interval was further divided into an ‘early prodromal phase’ (days −14 to −8) and a “late prodromal phase” (days −7 to −1), stratified by episode polarity. This structure is based on previous research^22,31,33^ and enables the identification of temporal dynamics surrounding episode onset. To verify our phases, we compared clinician- and self-rated symptoms across phases. Accordingly, euthymia showed minimal symptom burden (YMRS: 1.06 ± 1.88; self-rating: 49.88 ± 10.46). In contrast, depressive phases showed low YMRS and self-rated mood, while manic phases exhibited elevated scores (e.g., YMRS first week: 0.54 ± 1.24 vs 9.57 ± 5.07; self-rating first week: 39.71 ± 14.60 vs 54.88 ± 9.37). These results confirm that euthymia periods were stable and clearly distinct from symptomatic phases. See Table S1 for full details. The study was conducted in accordance with the Declaration of Helsinki and approved by the Ethics Committee of the Technical University of Dresden (EK-Nr.: 26012014). All participants provided written informed consent. Participants received a study smartphone (optional) and a monthly reimbursement of €35.

### Data acquisition and pre-processing

**Fig. 1:**
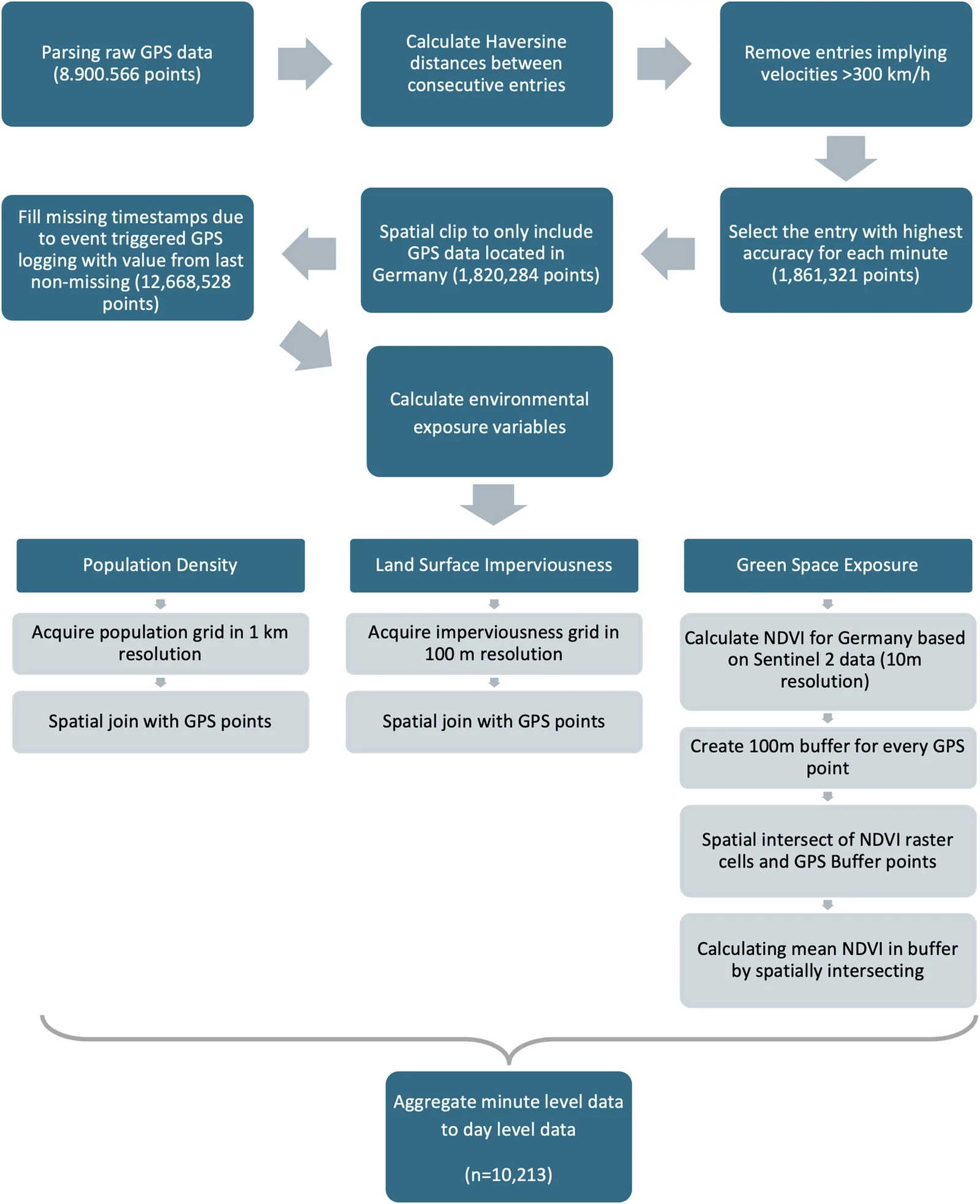
Data processing chart: overview of the GPS data processing methodology. Raw GPS data were cleaned, spatially restricted to Germany, and temporally regularized to one-minute intervals. Missing timestamps were imputed, and population density, land surface imperviousness as well as green space exposure were derived by linking GPS points to population density, land surface imperviousness, and green space exposure (NDVI). Minute-level data were aggregated to daily values for analysis.

Figure 1 provides a complete graphical overview of the data processing pipeline. Digital phenotyping was conducted using the movisensXS app (movisens GmbH, Karlsruhe, Germany), which collected both active and passive data. Alongside standard e-diary entries on mood, sleep, and medication use (adapted from the validated ChronoRecord system)^34,35^, we focused on GPS based location data. The initial sample comprised 31 participants; one dropped out and one was excluded due to data issues, leaving 29 with ≥10 months of data. High-resolution geospatial recordings ( ~ 5–10 s) yielded 8,900,566 data points. First, distances between consecutive points were then calculated using the Haversine formula. Entries implying speeds >300 km/h were excluded to remove likely GPS inaccuracies (e.g., due to GSM fallback). To improve computational efficiency, data were downsampled to one point per minute, yielding 1,820,284 entries. Additionally, we spatially restricted the dataset to the territory of Germany, as data on green spaces, population density, and imperviousness were only available for this region. GPS data collected via movisensXS are event-triggered to optimize energy efficiency, such that location points are only recorded when spatial movement occurs. Consequently, periods without movement (e.g., when participants remain at home) yield no entries. To enable calculation of the proportion of minutes spent in specific locations per day, missing data were forward-filled using the last observed location, thereby reconstructing complete 1440-min daily records. Following this procedure, the dataset comprised 12,668,528 entries.

### Measures–quantification of environment exposure

We quantified participants’ environmental context through measures of population density, impervious land cover, and surrounding green space. Population density (inhabitants/km²) was obtained from the 2011 German census at 1 km² resolution^36^. Information on land surface imperviousness, defined as the proportion of sealed or built-up surfaces relative to the total area in percent, was derived from the Copernicus HRL Imperviousness dataset at 100 m resolution^37^. For alignment with the BipoSense study period, the 2015 dataset was selected. To quantify green space exposure, defined as the proportion of vegetated land cover accessible within the immediate surroundings of each geolocation point, we employed Sentinel-2 satellite data due to its high spatial resolution (10 m) and broad geographical coverage, which is well suited to the nationwide scope of the BipoSense dataset. Sentinel-2 data became available in mid-2017 and provide reflectance information across several spectral bands (Bands 2–4 and 8: blue, green, red, and near-infrared). Specifically, we used satellite image data from 01.04.2022 to 30.09.2022. Green space analysis was conducted using Google Earth Engine (GEE), in which a 100-m buffer was applied around each geolocation point. Within this buffer, the Normalized Difference Vegetation Index (NDVI) was calculated using the formula:

1 $${NDVI}=\frac{{NIR}-{RED}}{{NIR}+{RED}}\,$$

NDVI values range from –1 to +1, with values > 0.5 indicating dense, healthy vegetation. In an additional step, we calculated the proportion of green space per location based on the threshold of NDVI > 0.5 within the defined buffer. In our analysis, we distinguished between the quality of surrounding green spaces, captured through NDVI values, and the quantity of green spaces, operationalized as the proportion of area with NDVI > 0.5.

**Fig. 2:**
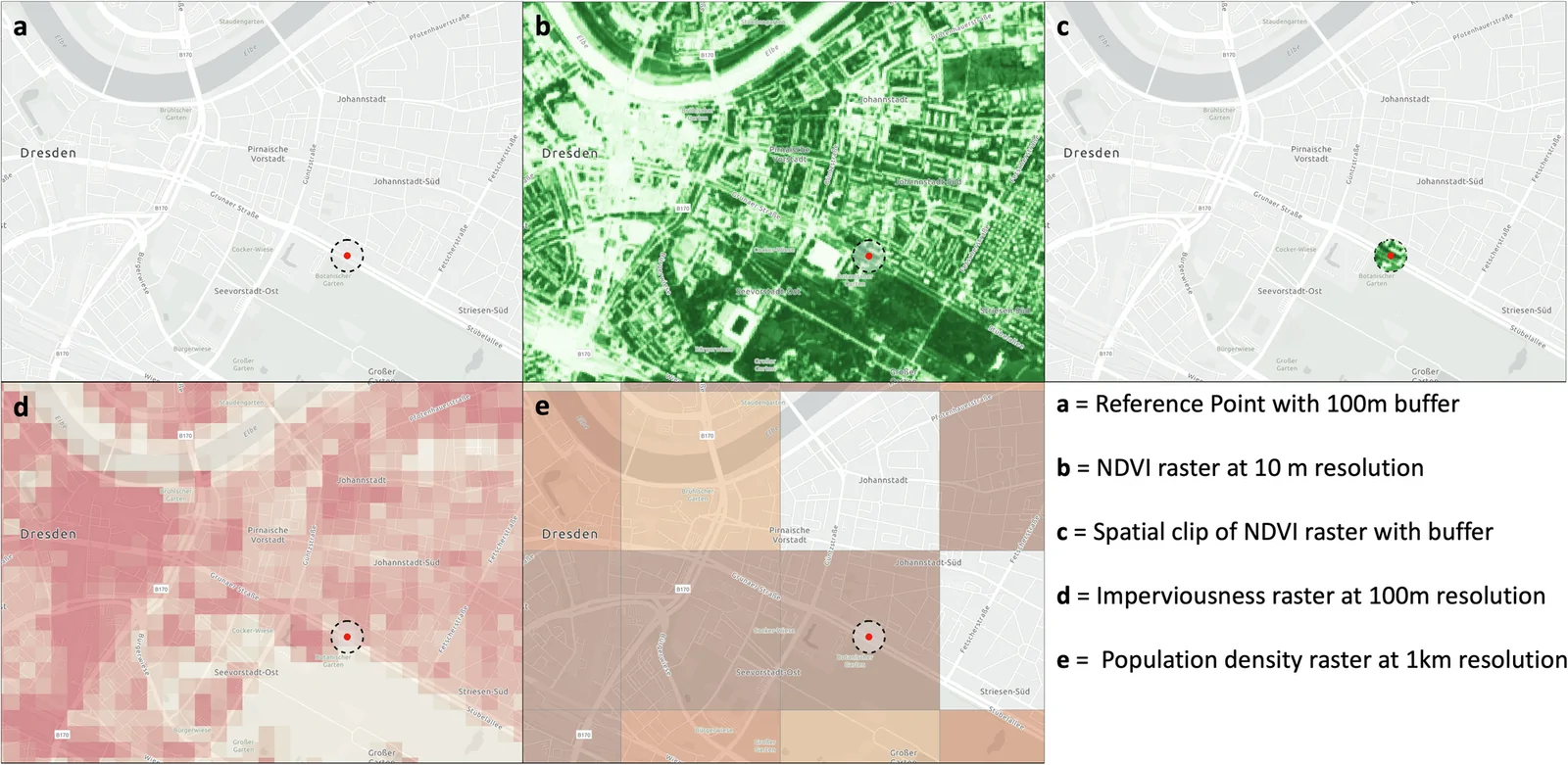
Spatial representation of environmental variables around a reference point in Dresden, Germany. **a** Reference point with a 100 m buffer (dashed line). **b** Normalized Difference Vegetation Index (NDVI) at 10 m resolution. **c** NDVI raster spatially clipped to the buffer. **d** Imperviousness raster at 100 m resolution. **e** Population density raster at 1 km resolution. The red circle denotes the reference location across all panels.

In the final step, all derived spatial variables were linked to the GPS data via a spatial join. Put simply, if a GPS point fell within a given polygon (e.g., a satellite-derived raster cell), the corresponding value was assigned to that point. Data were aggregated to daily means. Ultimately, this procedure yielded the following variables, which are also illustrated in Fig. 2:

- **Mean Population Density**: Average population count within 1 km raster cells based on the 2011 German census.
- **Mean Imperviousness**: Mean percentage of impervious surfaces (e.g., concrete, asphalt) within the 100 m raster cell surrounding each EMA GPS point.
- **Mean Green Area**: Proportion of green space (defined as NDVI > 0.5) within a 100 m buffer around each GPS measurement point.
- **Mean NDVI**: Average NDVI value within a 100 m buffer around the participant’s GPS locations, indicating vegetation density.

For each variable, variance was computed using a 14-day moving window. We selected a 14-day window to align with the SCID-based clinician interviews, which retrospectively cover the preceding 14 days and formed the basis for our phase definitions. For each day *k*, variance was calculated from the preceding 14 days including the indexed day (k–13 to *k*). Thus, the exposure window partially overlaps with the day for which affective status was modeled. We expect this overlap to be negligible in practice because it constitutes only 1 of 14 days in the window and because affective states were derived from clinician interviews that retrospectively covered a 14-day interval rather than reflecting a purely momentary state at day *k*. Missing values were not imputed; if fewer than seven valid data points were available, variance was set to missing. To reduce skewness, +1 was added to all values prior to applying the natural logarithm, yielding LNVAR. Moving averages were calculated similarly, as the arithmetic mean of observed values within the same 14-day window. All variables were rescaled to a 0–100 range and within-subject centered to account for inter-individual differences. Together, moving averages and variance allowed us to model not only whether participants were exposed to more or less urban or green environments, but also whether their exposure patterns were consistent or variable in the days preceding different affective states.

### Statistical analysis

To account for the hierarchical structure of the data, we used generalized linear mixed models (multilevel logistic regression) with fixed effects for each exposure parameter and random intercepts for participants to account for between-subject heterogeneity. Euthymia served as the reference category in all models. Variance and moving average of each nature exposure parameter were included as fixed main effects, and mood status (early/late pre-episode week or first/second/ongoing weeks vs. euthymia) was modeled as the binary outcome. We controlled the family-wise error rate using the Holm–Bonferroni method, applied separately for each mood status (depression: *n* = 40 models, (hypo)mania: *n* = 32), where n is the product of spatial predictors (4), metrics (2: moving average and variance), and outcome domains. These numbers result from the combination of *four spatial predictors* (population density, imperviousness, green area, NDVI), *two statistical metrics* (moving average and variance), and the *number of episode phases contrasted with euthymia* (five in depression, four in (hypo)mania). Thus, each model tested a single predictor–metric combination against one outcome domain.

To complement the statistical models and assess the practical, clinical relevance of the observed associations, we further evaluated the predictive performance of each environmental exposure parameter using receiver operating characteristic (ROC) curves. This approach allows us to quantify the sensitivity and specificity of predictors in distinguishing prodromal and episode phases from euthymia, providing insight into their potential utility for early detection in real-world settings. In our plots, the x-axis represents the false positive rate (1 – specificity), while the y-axis represents the true positive rate (sensitivity). A diagonal 45-degree line indicates chance-level prediction (AUC = 0.5), where sensitivity equals the false positive rate, meaning that any predictor along this line performs no better than random guessing. Following previous work^22^, a typical target area for clinical applicability can be defined as follows: a sensitivity of at least 50%, ensuring detection of at least every second emerging episode, and a specificity of at least 95%, minimizing false alarms. In our sample, this specificity corresponded approximately to one false alarm per month; however, this estimate depends on episode prevalence and overall predictive accuracy and may not generalize to other clinical settings.

Data processing, statistical analysis and visualization were conducted using the Julia programming language^38^ using the packages DataFrames.jl^39^, GeoStats.jl^40^, MixedModels.jl^41^ and Makie.jl^42^.

## Results

### Sample characteristics

Our final sample contained 29 patients (17 female; mean age = 43.8 ± 12.1 years; range 25–70). Sixteen were diagnosed with BD-I and twelve with BD-II. Over the one-year study period, 10,584 patient days were recorded, with high compliance: 97% of biweekly clinical interviews, 89% of daily e-diary entries, and 99% of passive sensing data were completed^39^. The dataset included 30 depressive and 20 (hypo)manic episodes, alongside 8763 euthymic days. Across all participants, 866 days were classified as depressive phases, comprising 161 early prodromal days, 164 late prodromal days, 179 episode-onset days, 168 days within a second depressive episode, and 194 days of ongoing depressive states. For (hypo)mania, 665 days were classified, including 133 early prodromal days, 141 late prodromal days, 147 episode-onset days, 146 days within a second (hypo)manic episode, and 98 days of ongoing (hypo)manic states. Additionally, 856 days were classified as missing. Extended (hypo)manic episodes lasting >3 weeks were excluded from statistical analyses because of insufficient recorded days.

Tables 1 and 2 present results of multilevel logit models comparing environmental exposure (moving averages and variance) between affective episodes, their prodromes and euthymia. Predictors are listed on the left-hand side. For (hypo)mania (Table 1), four columns display moving averages for the four outcomes: early prodromal phase, late prodromal phase, first (hypo)manic week, and second week (hypo)manic week. The four columns on the right-hand side show variance measures across the same weeks. Extended (hypo)manic episodes ( > 3 weeks) were excluded from analyses due to insufficient data density^30^. In the same manner, depressive outcomes are shown in Table 2. All environmental predictors were standardized to a 0–100 scale, enabling direct comparison of odds ratios across exposure domains.

**Table 1:** Multilevel logit models using variance and moving averages as predictors for disorder status in (hypo)mania

(Hypo)mania vs Euthymia								
	14- day moving averages				14-day moving variance (log-transformed)			
Disorder Status	Early prodromal	Late prodromal	1st week (hypo)manic episode	2nd week (hypo)manic episode	Early prodromal	Late prodromal	1st week (hypo)manic episode	2nd week (hypo)manic episode
	OR / p	OR / p	OR / p	OR / p	OR / p	OR / p	OR / p	OR / p
Green Area	0.951 / 0.003	0.963 / 0.064	0.977 / 0.295	0.964 / 0.027	1.478 / <0.001	1.435 / <0.001	1.348 / <0.001	1.641 / <0.001
NDVI	0.846 / 0.003	0.857 / 0.013	0.897 / 0.051	0.854 / 0.002	1.936 / <0.001	1.950 / <0.001	1.882 / < 0.001	2.388 / <0.001
Population Density	1.028 / 1.0	0.921 / 0.269	1.017 / 1.0	1.087 / 0.247	0.773 / 0.250	0.71 / 0.247	1.389 / 0.047	1.107 / 1.0
Impervious-ness	1.147 / 1.0	0.923 / 1.0	1.358 / 0.068	1.582 / 0.002	1.291 / 0.339	1.242 / 0.586	1.549 / < 0.001	1.573 / < 0.001

Predictors are presented in the left-hand column, while the first five columns display results for moving averages and the next five columns show variance measures across the respective episode phases compared with euthymia. To allow direct comparison of effect sizes across environmental indicators, all exposure variables were rescaled to a common 1–100 range; odds ratios therefore reflect comparable relative changes across predictors.b: Estimated unstandardized regression coefficients OR: Odds ratio P-values are adjusted for multiple testing for depression and (hypo)mania separately via Bonferroni-Holm correction; *P* values < 0.05 are highlighted in bold.

**Table 2:** Multilevel logit models comparing variance and moving averages as predictors for disorder status in depression

Depression vs Euthymia										
	14- day moving averages					14-day moving variance (log-transformed)				
Disorder Status	Early prodromal	Late prodromal	1st week depressive episode	2nd week depressive episode	Ongoing depressive weeks	Early prodromal	Late prodromal	1st week depressive episode	2nd week depressive episode	Ongoing depressive weeks
	**OR / p**	**OR / p**	**OR / p**	**OR / p**	**OR / p**	**OR / p**	**OR / p**	**OR / p**	**OR / p**	**OR / p**
Green Area	0.997 / 1.0	1.016 / 1.0	1.032 / < **0.007**	1.018 / 1.0	0.929 / < **0.001**	0.927 / 1.0	0.930 / 1.0	0.997 / 1.0	0.929 / 1.0	0.852 / **0.024**
NDVI	0.968 / 1.0	1.041 / 1.0	1.113 / < **0.004**	1.065 / 0.862	0.794 / < **0.001**	0.923 / 1.0	1.001 / 1.0	1.071 / 1.0	0.977 / 1.0	0.838 / 0.862
Population Density	1.040 / 1.0	1.014 / 1.0	1.001 / 1.0	0.999 / 1.0	1.004 / 1.0	0.642 / < **0.001**	0.742 / **0.048**	0.792 / 0.105	0.787 / 0.150	0.926 / 1.0
Impervious-ness	0.987 / 1.0	0.930 / 1.0	0.834 / 0.560	0.810 / 0.427	0.526 / **0.004**	1.169 / 0.847	1.071 / 1.0	0.827 / 1.0	0.709 / 0.111	0.676 / **0.023**

Predictors are presented in the left-hand column, while the first five columns display results for moving averages and the next five columns show variance measures across the respective episode phases compared with euthymia. To allow direct comparison of effect sizes across environmental indicators, all exposure variables were rescaled to a common 1–100 range; odds ratios therefore reflect comparable relative changes across predictors.b: Estimated unstandardized regression coefficients OR: Odds ratio P-values are adjusted for multiple testing for depression and (hypo)mania separately via Bonferroni-Holm correction; *P* values < 0.05 are highlighted in bold.

Hypothesis 1) Moving Averages

Consistent with our first hypothesis for (hypo-mania), the early prodromal week of (hypo)manic episodes (table 1; left-hand side) was significantly associated with the moving averages of green area (OR = 0.951, *p* = 0.003) and NDVI (OR = 0.846, *p* = 0.003). Odds below 1 indicated that less green area exposure was related to the occurrence of a prodromal week. During the late prodromal phase of (hypo)manic episodes, green area exhibited a trend (OR = 0.963, *p* = 0.064), while NDVI was statistically significant (OR = 0.857, *p* = 0.013), suggesting that diminished green space exposure is also predictive for the late prodromal week. However, for depressive prodromal phases (table 2; left-hand side) we found no significant associations after rigorous Bonferroni-Holm correction.

Building on these findings, we explored environmental signatures during acute and ongoing episodes. (Hypo)manic episode weeks (table 1; left-hand side) were partly predicted by moving averages of green space (e.g., NDVI OR = 0.854, *p* = 0.002 in second week), with odds ratios below 1.0 pointing to reduced green space exposure. Depressive episode weeks (Table 2; left-hand side) were predicted by green area, but only for the first (green *p* < 0.007; NDVI *p* < 0.004) and the ongoing week (green area: *p* < 0.001; NDVI *p* < 0.001) and not for the 2nd depressive week. Most interestingly, ORs reversed over time with first week showing ORs above 1 (green area: OR = 1.032; NDVI OR = 1.113) and ongoing weeks revealing ORs below 1 (green area: OR = 0.929; NDVI OR = 0.794).

Second, we explored whether other environmental indicators, including population density and exposure to impervious surfaces, might provide additional insights. Associations were generally weak across prodromal and episode phases, with only imperviousness showing a significant predictive effect on the second week of (hypo)manic episodes (OR = 1.582, *p* = 0.002). ORs above 1 reveal that more time spent in highly impervious urban areas are predictive for manic episode. In contrast, ongoing depressive weeks were significantly predicted by reduced imperviousness exposure (OR = 0.526, p = 0.004), i.e. reduced engagement with highly built-up areas.

Hypothesis 2) Variance measures

Findings from hypothesis 2, namely whether increased intra-individual variance of green space exposure is predictive for prodromal phases, as an early warning sign according to DST, are depicted in the right-hand side of Tables 1 and 2. For (hypo)manic episodes, elevated variance significantly predicted both early and late prodromal phases. Specifically, higher variability in time spent in green areas predicted (hypo)manic status in the early prodromal (OR = 1.478, *p* < 0.001) and late prodromal phase (OR = 1.435, *p* < 0.001). NDVI variance showed a similar pattern, with elevated variability predicting the early (OR = 1.936, *p* < 0.001) and late prodrome (OR = 1.950, *p* < 0.001), suggesting that fluctuations in greenness exposure preceded the onset of manic episodes. For depressive episodes, variance in green space exposures was, in contrary, not significantly predicting prodromal weeks.

In our additional exploratory analysis, we investigated whether these variance patterns persisted into acute and ongoing episode weeks and whether other environmental indicators might reveal additional insights. For (hypo)manic episodes, the variance of green area remained a statistically significant predictor for the first (OR = 1.348, *p* < 0.001) and second week (OR = 1.641, *p* < 0.001). Similarly, NDVI variance was still predictive with ORs above 1.0 (first week: OR = 1.882, *p* < 0.001; second week: OR = 2.388, *p* < 0.001), suggesting that heightened fluctuations in green space exposure predicts beyond prodromal phases. For depressive episodes variance indices showed, again, limited predictive value.

Exploratory analyses of the other environmental factors revealed modest signals. The variance of population density was predictive only for the first (hypo)manic week, while imperviousness variance was predictive for the first (OR = 1.549, *p* < 0.001) and second (hypo)manic week (OR = 1.573, *p* < 0.001). For depressive episodes, lower variance in population density emerged as an early-warning signal during early (OR = 0.642, *p* < 0.001) and late prodromal phases (OR = 0.742, *p* = 0.048), and a decreased variance of imperviousness (OR = 0.676, *p* = 0.023) predicted ongoing depressive episodes.

### Clinical relevance of the observed parameters

**Fig. 3:**
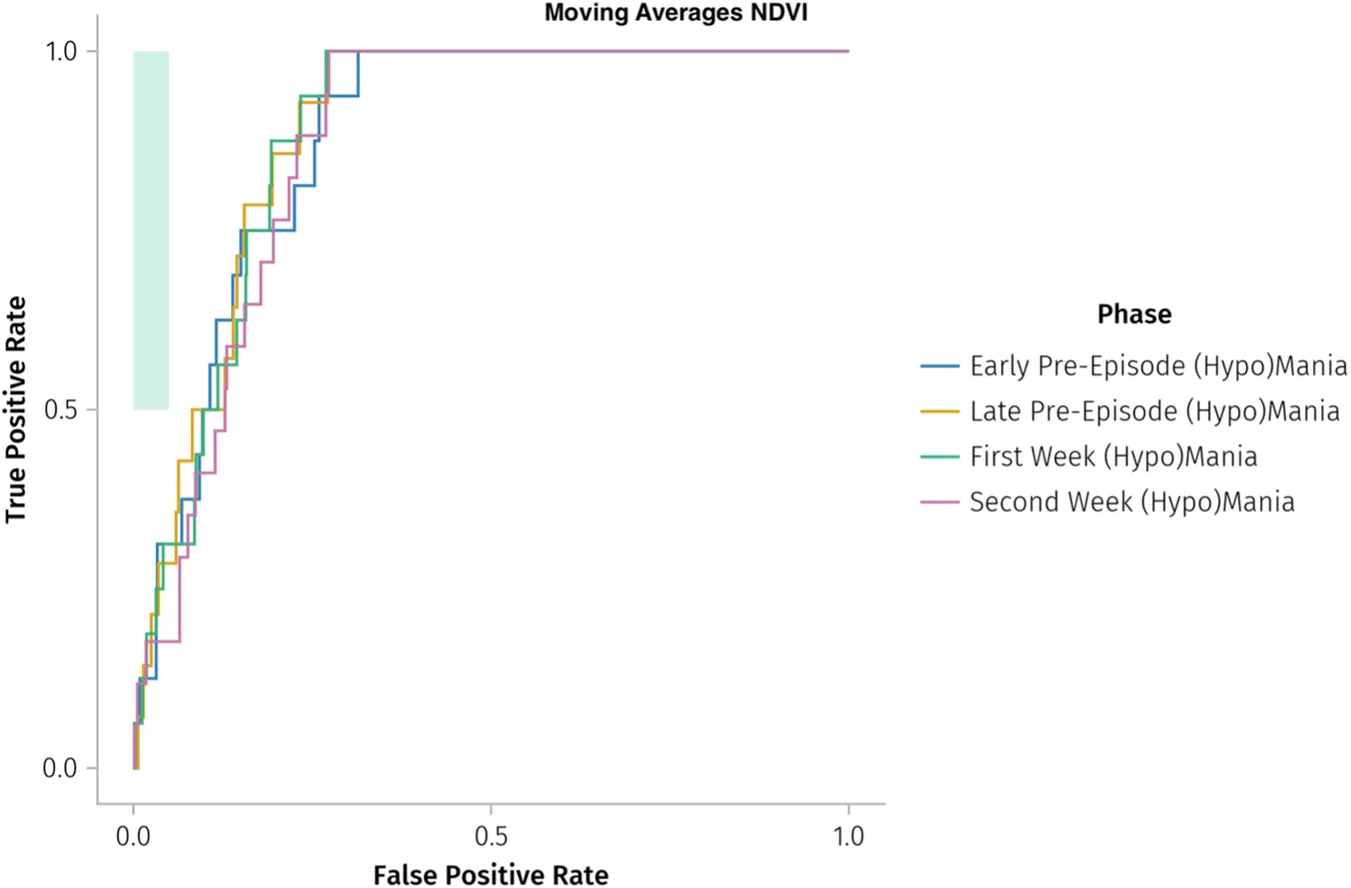
Predictive performance of moving average in NDVI in anticipating manic episodes. ROC curves showing the ability of mean NDVI to predict different phases of (hypo)mania. The x-axis represents the false positive rate, and the y-axis the true positive rate. Curves near the top-left indicate higher accuracy. Colors indicate phases: blue–Early Pre-Episode (Hypo)Mania, yellow–Late Pre-Episode (Hypo)Mania, green–First Week (Hypo)Mania, pink–Second Week (Hypo)Mania. Additionally, the turquoise shaded area indicates the combination of high sensitivity ( > 50%) and high specificity ( > 95%) required for clinical applicability.

**Fig. 4:**
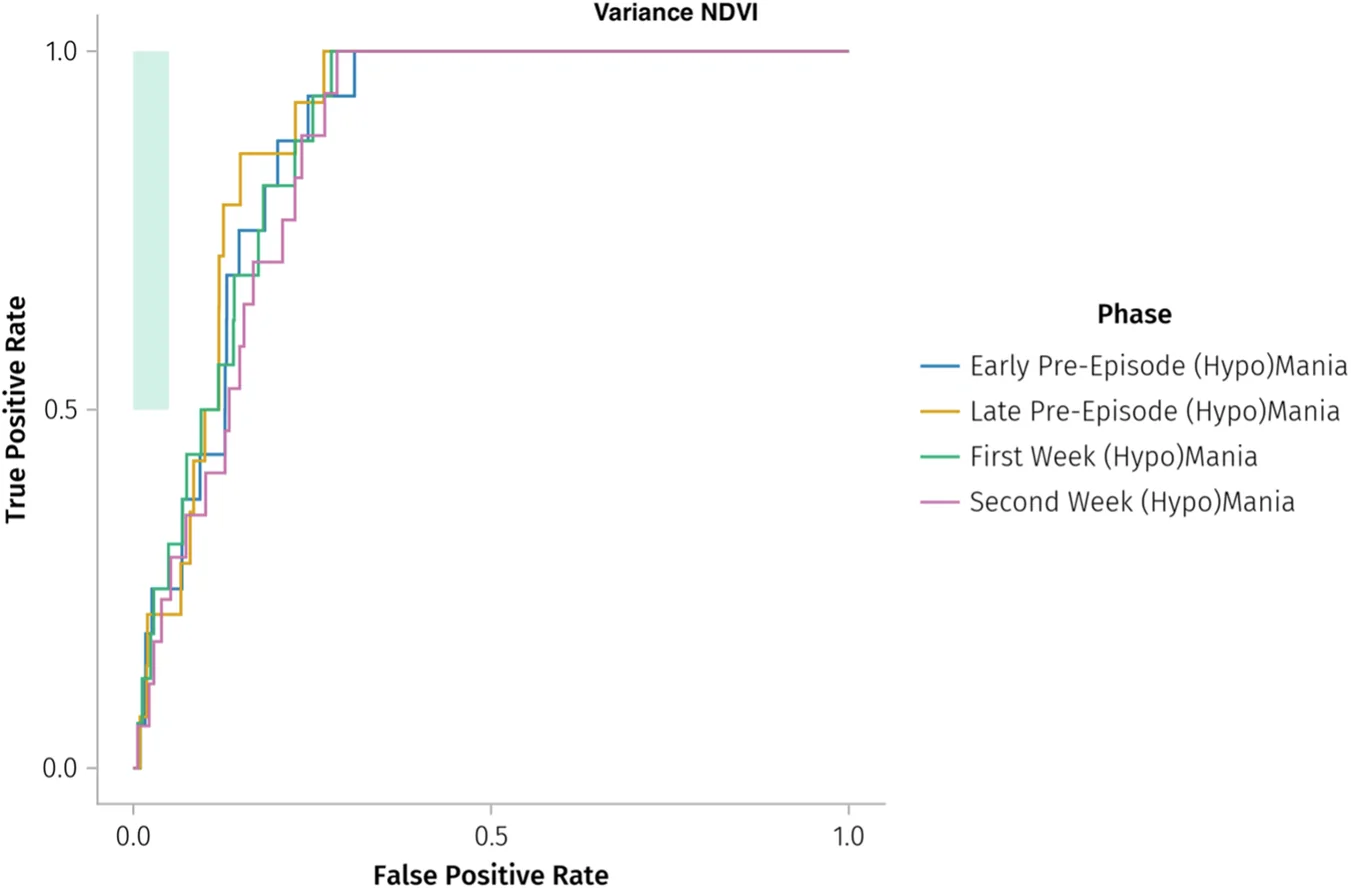
Predictive performance of variance in NDVI in anticipating manic episodes. ROC curves showing the ability of variance in NDVI to predict different phases of (hypo)mania. The x-axis represents the false positive rate, and the y-axis the true positive rate. Curves near the top-left indicate higher accuracy. Colors indicate phases: blue–Early Pre-Episode (Hypo)Mania, yellow–Late Pre-Episode (Hypo)Mania, green–First Week (Hypo)Mania, pink–Second Week (Hypo)Mania. Additionally, the turquoise shaded area indicates the combination of high sensitivity ( > 50%) and high specificity ( > 95%) required for clinical applicability.

To assess the clinical relevance of the observed predictors, we chose NDVI as the most consistent greenness predictor and computed ROC curves, depicted in Figs. 3 and 4. The target region for clinical applicability is indicated by a turquoise shaded area, highlighting the combination of high sensitivity ( > 50%) and high specificity ( > 95%) required for practical use^22^. Across all time windows (early prodromal, late prodromal, first week, and second week of (hypo)mania) the predictors consistently exceeded chance-level performance (AUC > 0.5), demonstrating their potential utility in detecting manic episodes. However, while the AUC values suggest good discriminatory power, the curves generally do not enter the turquoise target region. This indicates that, although NDVI shows promise as a digital phenotyping marker, its current predictive performance still generates too many false alarms or misses episodes to be considered reliably actionable in a clinical context. The strongest signals appear during the late and early prodromal periods. Additional ROC plots illustrating 14-day moving averages and 14-day moving variance for green space exposure, population density, and imperviousness are provided in the Supplementary Materials (Figs. S1 and S2).

## Discussion

Using high-resolution GPS data from 29 individuals with BD over 12 months, we quantified minute-level exposure to green space (amount and quality), population density, and impervious surfaces and related them to emerging and ongoing affective episodes. Day-level mood states were regressed on moving averages and variance metrics via multilevel logistic models.

Partially consistent with our first hypothesis, reduced green space exposure, i.e., green area and NDVI, significantly predicted early and late prodromal phases transitioning into (hypo)mania. This finding is in line with the assumption that green space exerts a stress-buffering effect. Reduced engagement with natural environments may contribute to the emergence of (hypo)manic symptoms. In contrast, moving averages of our green space exposure variables did not significantly predict prodromal depressive episode weeks, indicating that Hypothesis 1 was not supported for depression. One possible explanation is that depressive prodromes and episodes are associated with reduced activity and mobility, which may reduce day-to-day variability in location-based exposures and thereby limit the detectability of changes in green space engagement^43^.

Our exploratory analyses indicated that reduced green space exposure persisted as a significant predictor into (hypo)manic episodes, while for depression, ORs of green exposure initially increased but attenuated or reversed during ongoing weeks. Other environmental factors showed weaker effects suggesting that it is not urbanicity per se, but rather the presence (or absence) of green space itself, which can occur even within highly urbanized environments such as parks or recreational areas.

Consistent with Hypothesis 2, which was theoretically grounded in DST and explicitly predicted increased within-person variability in green space exposure prior to episode onset, higher 14-day moving variance in green area and NDVI predicted prodromal phases of (hypo)manic episodes. These fluctuations appear to reflect changes in individuals’ behavioral patterns and engagement with their environment, consistent with theoretical predictions that rising variance can signal imminent critical transitions. For depressive episodes, however, we did not find support for Hypothesis 2, as variance in green space exposure was not a reliable predictor of depressive prodromal weeks, suggesting that the prodromal dynamics of depression may involve other dimensions of behavior or context.

Exploratory analyses of other environmental indicators revealed complementary insights. For (hypo)manic episodes, elevated variance as a predictor continued into acute weeks in both green area and NDVI, suggesting that fluctuations in green space exposure reflect ongoing destabilization beyond prodromes. Population density and imperviousness, while not predictive during prodromes, also showed increased variance as predictive during early manic weeks, indicating that broader environmental instability may accompany emergent manic states. In contrast, depressive episodes exhibited almost no significant variance predictors.

Collectively, these findings suggest that increased variance in green space exposure is consistent with DST-style early-warning dynamics for (hypo)mania, while appearing less informative for depression. Additionally, fluctuations in environmental exposure during manic episodes appear not to be limited to green space, but also involve urban features such as population density and impervious surfaces.

However, several limitations of our analysis warrant consideration. First, although this study extends the temporal resolution and duration of previous investigations^20,21^ and included participants with a high number of prior affective episodes, the absolute number of newly emerging episodes remained limited. Future studies aiming to predict emerging episodes may benefit from both longer observation periods, such as the 18-month duration implemented in a recent randomized controlled trial^18^ as well as and larger sample sizes.

Second, our variables (e.g., green space coverage or population density within a defined radius) are only proxies for the individual experience of the environment. For example, a participant living in a high-rise building might have a view blocked by neighboring buildings, limiting their actual exposure to nearby green space despite the high coverage indicated by GIS-derived metrics. Thus, individual perception and interaction with the environment depend on additional factors that are difficult to capture with area-level proxies, introducing inherent imprecision to our approach. However, GIS-derived metrics of green space definitely index a heightened probability to be visually exposed to green space.

Third, the temporal alignment between environmental data and GPS recordings was not perfect. For example, population density was derived from the 2011 German census, whereas the next nationwide raster dataset was only available in 2022, while participant monitoring took place over a 12-month period from November 2014 to June 2016. Although minor discrepancies between the measured exposure and the actual environment at the time of observation cannot be ruled out, these datasets represent the best available approximations.

Additionally, our 14-day exposure metrics (moving average and log-variance) were computed using a rolling window that included the indexed day (*t* − *13* to *t*). This creates a small degree of temporal overlap between exposure and the day-level affective status used in the models and therefore limits a strict interpretation in terms of purely prospective prediction. Because emerging mood symptoms may influence mobility and thereby environmental exposure, part of the observed associations may reflect contemporaneous coupling rather than unidirectional effects. We expect the magnitude of this overlap to be small because it affects only 1 of 14 days in the window and because clinician ratings summarize symptom status retrospectively over a 14-day interval rather than capturing an immediate state at day *t*.

Finally, participants were aware that their GPS data were being collected, which may have influenced their behavior. However, since we covered an extended observation period of 365 days, we do not assume that participants consistently altered their behavior throughout the entire duration.

Building on our findings, future work should move beyond purely behavioral and spatial signatures towards mechanistic models that link green space exposure to neurobiological processes in BD. There are already several hypotheses about how mechanisms could look like: Green space exposure may promote hypothalamic-pituitary-adrenal (HPA) axis stabilization, thereby reducing cortisol levels and modulating stress-related neuroinflammation^44,45^. Chronic HPA activation is implicated in BD pathophysiology, and attenuation of this response may reduce affective instability. Contact with green environments may support neuroplasticity and gray matter preservation in regions such as the prefrontal cortex and hippocampus^46^, which are frequently altered in BD^47^. Additionally, green spaces may strengthen immune function and reduce pro-inflammatory cytokine production, both of which are implicated in the neuroimmune dysregulation observed in BD patients^48,49^. Furthermore, green spaces may promote restorative cognitive and emotional processes, enhance executive function, and improve emotion regulation through visual and multisensory stimuli that engage neural circuits associated with attention and mood regulation^50,51^.

Affective episode onset in BD is likely driven by the interplay of multiple environmental, behavioral, and clinical processes. Future work should therefore prioritize multivariate and machine-learning–based modeling approaches that integrate spatial exposures with additional digital phenotypes, such as mobility dynamics, circadian regularity, sleep, or physiological stress markers. Such integrative frameworks have the potential to substantially improve predictive accuracy and advance the clinical utility of passive sensing approaches.

In sum, our work advances prior research by integrating location-based passive sensing with intensive longitudinal methods, demonstrating how interactions with green spaces can yield meaningful signals in BD. These findings lay the foundation for personalized interventions that leverage environmental context to enhance affective stability in BD.

## Supplementary information

NPJ_mentalhealth_supplementary_260706.

## Data Availability

The data are highly sensitive, as raw GPS data cannot be fully anonymized and therefore cannot be publicly shared. Aggregated data, which do not allow for the identification of individual participants, are available from the corresponding author upon reasonable request.

## Code availability

The complete code used for all analyses is freely available on GitHub (https://github.com/CarlBittendorf/BipoSense).
