# Supplementary material for: Green space exposure predicts manic episodes in bipolar disorder: one year longitudinal analysis using smartphone-derived GPS data
Source: Npj Ment Health Res. 2026 Sep 24;5:52. doi: 10.1038/s44184-026-00242-1 (PMC13612424; doi:10.1038/s44184-026-00242-1)
Supplement: Supplementary file 1 [file 44184_2026_242_MOESM1_ESM.pdf]

# Green space exposure predicts manic episodes in bipolar disorder: one year longitudinal analysis using smartphone-derived GPS data

Guth, M. [1]*, Bittendorf, C.A. [2], Krug, C. [3], Mühlbauer, E. [3], Severus, W.E. [4, 5], Bauer, M. [3], Reichert, M. [6,7] Ludwig, V.M. [3], & Ebner-Priemer, U. W.[2,7]

1) Faculty of Sport Science, Department of eHealth and Sports Analytics, Ruhr-University Bochum, Bochum, Germany

2) Mental mHealth Lab, Institute of Sports and Sports Science, Karlsruhe Institute of Technology, Karlsruhe, Germany.

3) Department of Psychiatry and Psychotherapy, University Hospital Carl Gustav Carus, Technical University Dresden, Dresden, Germany.

4) Faculty of Medicine Carl Gustav Carus, Technical University Dresden, Dresden, Germany

5) Asklepios Klinik Nord-Ochsenzoll, Hamburg, Germany.

6) Department of Sport and Exercise Science, Faculty of Natural and Life Sciences, University of Salzburg, Salzburg, Austria.

7) Department of Psychiatry and Psychotherapy, Central Institute of Mental Health, University of Heidelberg, Medical Faculty Mannheim, Mannheim, Germany.

*Corresponding author

# Supplementary Material

a. Clinical and Self-Rated Validation of Phase Definitions

**Table S1: Mean (± SD) Scores of Clinician-Rated and Self-Rated Measures Across Baseline and the Eleven Mood Phases**

| PHASE | YMRST MEAN | BRMRS MEAN | MADRS MEAN | CGI-S BD MEAN | SELF-RATING MEAN | YMRS SD | BRMRS SD | MADRS SD | CGI-S BD SD | SELF-RATING SD |
|---|---|---|---|---|---|---|---|---|---|---|
| EUTHYMIA | 1.06 | 0.49 | 4.26 | 1.37 | 49.89 | 1.88 | 1.13 | 5.31 | 0.70 | 10.46 |
| DEPRESSION EARLY PRODROMAL | 0.75 | 0.34 | 11.25 | 1.97 | 45.43 | 2.04 | 0.80 | 6.38 | 0.93 | 13.80 |
| DEPRESSION LATE PRODROMA | 0.78 | 0.28 | 11.76 | 1.86 | 42.89 | 2.03 | 0.77 | 6.91 | 0.92 | 14.77 |
| DEPRESSION FIRST WEEK | 0.54 | 0.14 | 19.84 | 2.29 | 39.71 | 1.24 | 0.57 | 8.55 | 1.51 | 14.60 |
| DEPRESSION SECOND WEEK | 0.59 | 0.15 | 19.86 | 2.36 | 35.27 | 1.28 | 0.59 | 8.66 | 1.52 | 14.82 |
| DEPRESSION ONGOING WEEKS | 0.61 | 0.11 | 24.90 | 2.13 | 31.14 | 1.08 | 0.32 | 10.62 | 1.68 | 16.31 |
| MANIA EARLY PRODROMAL | 3.44 | 1.75 | 5.89 | 1.87 | 51.90 | 2.79 | 1.74 | 6.43 | 0.87 | 9.43 |
| MANIA LATE PRODROMAL | 3.54 | 1.96 | 5.41 | 1.96 | 50.73 | 2.79 | 1.80 | 6.37 | 0.89 | 10.63 |
| MANIA FIRST WEEK | 9.57 | 5.33 | 4.52 | 3.00 | 54.88 | 5.07 | 2.74 | 5.82 | 1.12 | 9.37 |
| MANIA SECOND WEEK | 9.66 | 5.39 | 4.53 | 3.01 | 57.11 | 5.05 | 2.73 | 5.85 | 1.13 | 10.33 |
| MANIA ONGOING WEEKS | 9.98 | 5.32 | 3.92 | 3.25 | 51.24 | 4.42 | 2.22 | 4.49 | 1.03 | 10.40 |

b. ROC Plots

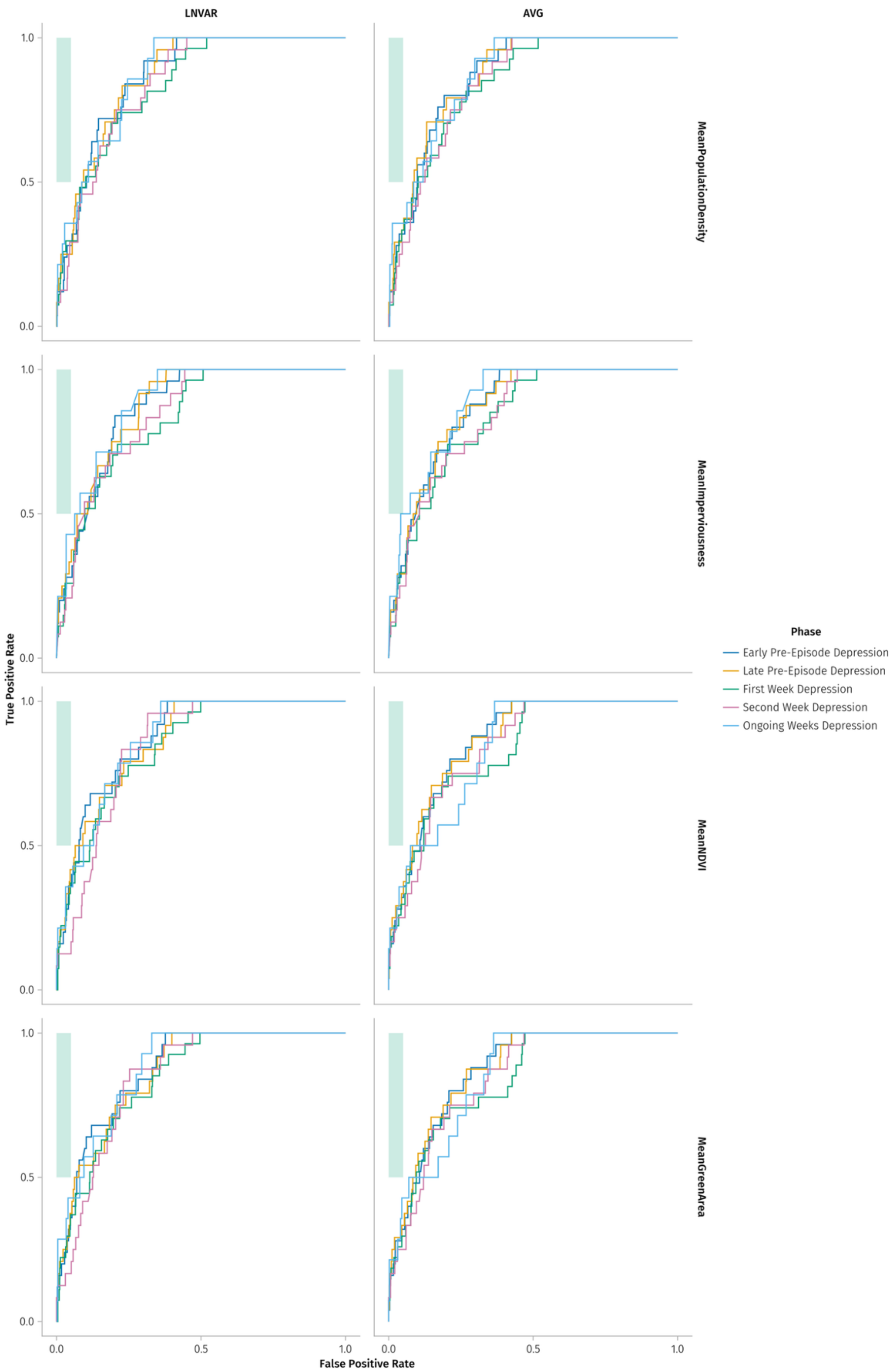


Figure S1: **Predictive performance of moving average and Variance for Populations Density, Imperviousness, NDVI and Greenarea in anticipating depressive episodes.** ROC curves show the ability to predict different phases of depression. The x-axis represents the false positive rate, and the y-axis the true positive rate. Curves near the top-left indicate higher accuracy. Colors indicate phases: dark blue – Early Pre-Episode depression, yellow – Late Pre-Episode depression, green – First Week depression, pink – Second Week depression, light blue - Ongoing Weeks Depression Additionally, the teal shaded area indicates the combination of high sensitivity (> 50%) and high specificity (> 95%) required for clinical applicability

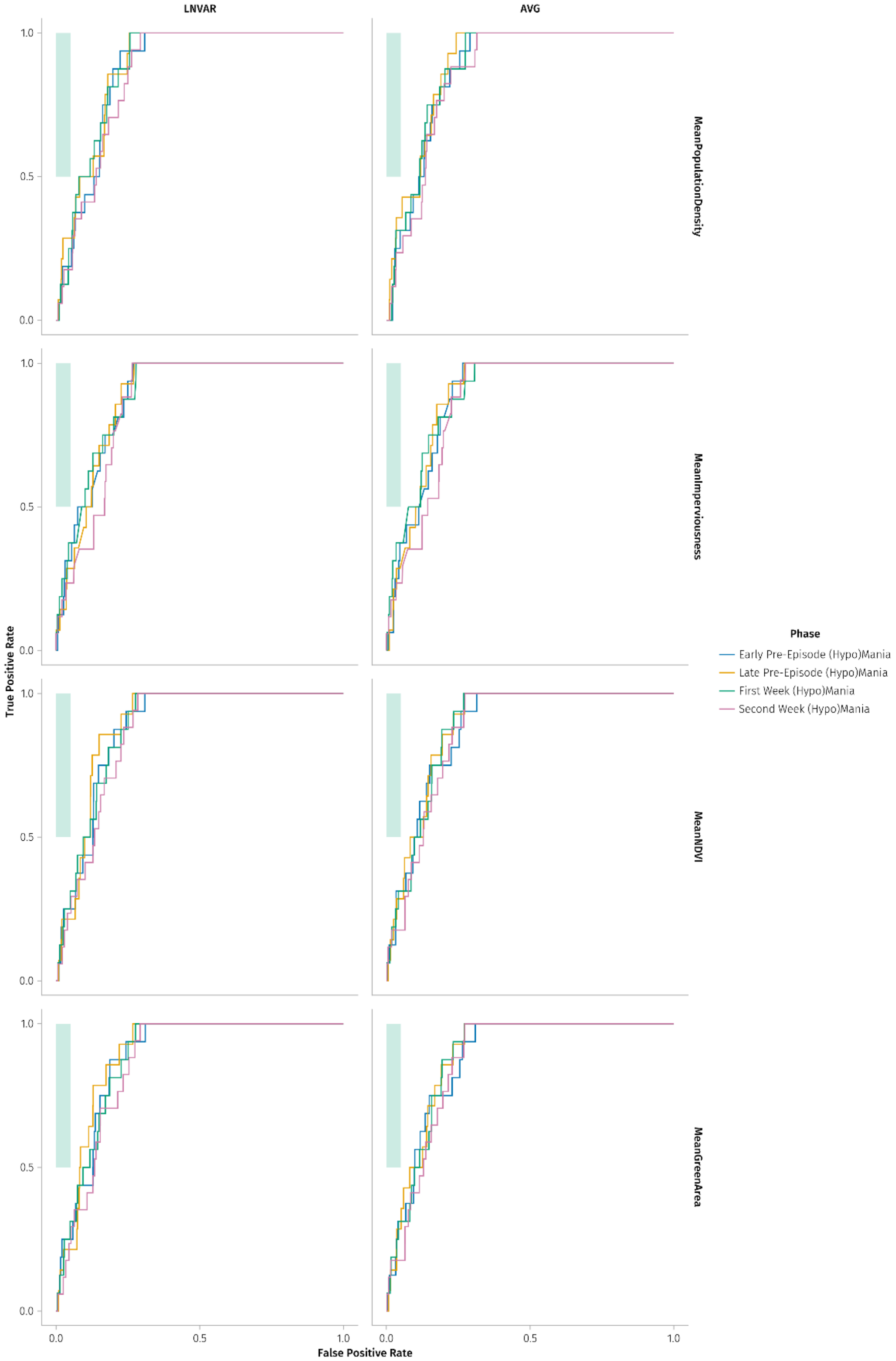


Figure S2: **Predictive performance of moving average and Variance for Populations Density, Imperviousness, NDVI and Greenarea in anticipating (hypo)manic episodes.** ROC curves show the ability to predict different phases of (hypo)mania. The x-axis represents the false positive rate, and the y-axis the true positive rate. Curves near the top-left indicate higher accuracy. Colors indicate phases: blue – Early Pre-Episode (Hypo)Mania, yellow – Late Pre-Episode (Hypo)Mania, green – First Week (Hypo)Mania, pink – Second Week (Hypo)Mania. Additionally, the blue-green shaded area indicates the combination of high sensitivity (> 50%) and high specificity (> 95%) required for clinical applicability
